# High dose simultaneous integrated boost for node positive cervical cancer

**DOI:** 10.1186/s13014-021-01818-1

**Published:** 2021-05-17

**Authors:** Iresha Jayatilakebanda, Yat Man Tsang, Peter Hoskin

**Affiliations:** 1 Mount Vernon Cancer Centre, Rickmansworth Road, Northwood, Middlesex, HA6 2RN UK; 2 University of Manchester, Manchester, UK

## Abstract

**Introduction:**

Lymph node metastases presenting with locally advanced cervical cancer are poor prognostic features. Modern radiotherapy approaches enable dose escalation to radiologically abnormal nodes. This study reports the results of a policy of a simultaneous integrated boost (SIB) in terms of treatment outcomes.

**Materials and methods:**

Patients treated with radical chemoradiation with weekly cisplatin for locally advanced cervical cancer including an SIB to radiologically abnormal lymph nodes were analysed. All patients received a dose of 45 Gy in 25 fractions and a SIB dose of 60 Gy in 25 fractions using intensity modulated radiotherapy/volumetric modulated arc therapy, followed by high dose rate brachytherapy of 28 Gy in 4 fractions. A control cohort with radiologically negative lymph nodes was used to compare impact of the SIB in node positive patients. Treatment outcomes were measured by overall survival (OS), post treatment tumour response and toxicities. The tumour response was based on cross sectional imaging at 3 and 12 months and recorded as local recurrence free survival (LRFS), regional recurrence free survival (RRFS) and distant recurrence free survival (DRFS).

**Results:**

In between January 2015 and June 2017, a total of 69 patients with a median follow up of 30.9 months (23 SIB patients and 46 control patients) were identified. The complete response rate at 3 months was 100% in the primary tumour and 83% in the nodal volume receiving SIB. The OS, LRFS, RRFS and DRFS at 3 years of the SIB cohort were 69%, 91%, 79% and 77% respectively. High doses can be delivered to regional pelvic lymph nodes using SIB without excessive toxicity.

**Conclusion:**

Using a SIB, a total dose of 60 Gy in 25 fractions chemoradiation can be delivered to radiologically abnormal pelvic nodes with no increase in toxicity compared to node negative patients. The adverse impact of positive nodal status may be negated by high dose deposition using SIB, but larger prospective studies are required to confirm this observation.

## Introduction

Cervical cancer is a common cancer and worldwide is the fourth most common cancer for both incidence and mortality in women [1]. In the United Kingdom incidence rates have been reduced due to the effective screening programmes [2], but still patients present with locally advanced disease and lymph node involvement.

For patients with locally advanced cervical cancer, concurrent cisplatin-based chemotherapy with external beam radiotherapy (EBCRT), followed by brachytherapy is considered to be the standard of care [3]. The prognosis for patients presenting with evidence of nodal metastases is substantially worse than those that have normal nodal anatomy on initial staging when treated conventionally [4]. Lymph node involvement was not formally addressed in the original FIGO staging. In the current FIGO staging (2018), patients with positive pelvic lymph nodes are classified as stage IIIC1 and those with positive para-aortic nodes are classified as stage IIIC2 [5]. With advances in technology, more accurate disease staging and diagnosis using 18F-fluoro-2-deoxy-D-glucose positron emission tomography (18FDGPET) and multiparametric magnetic resonance (MR) imaging are possible and have replaced the need for laparoscopic node evaluation [6].

Alongside these developments in diagnostic imaging, there has been rapid progress in the technology used for planning and delivery of radiotherapy including intensity modulated radiotherapy (IMRT), volumetric modulated arc therapy (VMAT) and image guided radiotherapy (IGRT) in the past decade. These new techniques are now commonly used, and they enable accurate delivery of therapeutic doses of radiation and simultaneous integrated boosts (SIB) to high doses in nodal chains for patients receiving cervical EBCRT [7]. Improved understanding of the distribution of nodal disease in cervical cancer and patterns of relapse has led to more risk adjusted protocols such as that defined in the EMBRACE II study [8, 9].

There is no consensus on the optimum external beam radiotherapy technique when there are positive pelvic lymph nodes [10]. Historically, patients with positive lymph node involvement were treated with concurrent chemo-radiotherapy of 45–50 Gy over 5 weeks followed by brachytherapy, with a sequential ‘top up’ boost to the positive lymph nodes delivering a further 5-8 Gy. Whilst these EBRT radiation doses with brachytherapy may be sufficient to eliminate the primary tumour, nodal control at such dose is uncertain. However whilst a higher radiotherapy dose to visible bulky nodal disease is assumed to result in better treatment outcomes the benefits of having a nodal EBRT boost on overall survival and progression free survival remain controversial [10].

The aim of this service evaluation study was to assess treating extended nodal volumes with a SIB up to 60 Gy to radiologically positive nodes in patients referred for radical chemoradiation, in terms of response, toxicity, recurrence free survival (RFS) and overall survival (OS).

## Materials and methods

Patients with locally advanced cervical cancer were offered radical concurrent chemoradiation incorporating SIB to radiologically positive pelvic or para-aortic lymph nodes. Those treated postoperatively or with atypical histology (clear cell or small cell) were excluded. In addition to routine demographic data, nodal site and size, dosimetry, treatment response and toxicity were extracted.

Treatment was standardised delivering a radiotherapy dose of 45 Gy in 25 daily fractions using IMRT/VMAT with weekly cisplatin 40 mg/m^2^ using the EMBRACE planning guidelines [8] for the primary site and nodal volumes defined by a standard nodal atlas [11]. The internal and external nodes, obturator, presacral and common iliac were included routinely extended to the level of the renal vessels if common iliac nodes were involved and the lower border of T10 if there were abnormal nodes above the aortic bifurcation. All patients were staged with a pelvic multiparametric MR scan and computed tomography (CT) scans of the chest, abdomen and pelvis. For patients showing equivocal node status on CT, ^18^FDGPET was performed. Radiologically abnormal lymph nodes were identified on the planning CT scans and a separate clinical target volume (CTV) defined which was expanded by 5 mm globally to form the planning target volume (PTV). This was always inside the 45 Gy nodal PTV and using a SIB all nodes in the node positive patients were boosted to 60 Gy in 25 fractions. Dose planning constraints are shown in Table 1. All patients subsequently received high dose rate brachytherapy delivering 28 Gy in 4 fractions over 3 days to the high-risk CTV as defined in the EMBRACE study [8]. Mean nodal dose (Dmean) to right and left pelvis was calculated by extracting the EBRT doses to the nodal PTV and brachytherapy doses to point B converting the dose to a 2 Gy equivalent dose (EQD2 calculation) using an alpha beta value of 10.

**Table 1:** Planning dose constraints

Structure	Parameter	Dose constraint
Bowel		
	V30Gy	100 cm^3^ With boost 250 cm^3^
	V40Gy	350 cm^3^ With boost 500 cm^3^
	Maximum dose	47.3 Gy With boost 60.0 Gy
Sigmoid		
	Maximum dose	47.3 Gy With boost 60.0 Gy
Bladder	V30Gy	85%
	V40Gy	75%
	Maximum dose	47.3 Gy With boost 60.0 Gy
Rectum	V30Gy	95%
	V40Gy	85%
	Maximum dose	47.3 Gy With boost 60.0 Gy
Spinal cord	Maximum dose	48 Gy
Lt Fem head	Maximum dose	50 Gy
Rt Fem head	Maximum dose	50 Gy

After completing radiotherapy, patients were assessed prospectively at 4 weeks, 12 weeks, 6 months and 6 monthly thereafter. Treatment outcomes were measured by post treatment tumour response, sites of recurrence, overall survival and toxicities. The tumour response was based on size criteria on CT thorax, abdomen and pelvis at 3 and 12 months and recorded as local recurrence free survival (LRFS), regional recurrence free survival (RRFS) and distant recurrence free survival (DRFS). Post treatment toxicities were graded using Common Terminology Criteria for Adverse Events (Version 4.0). Toxicity events are presented as the maximum toxicity reported at any follow-up time; acute toxicity was defined up to 12 weeks and late toxicity from 6 months onwards.

A control group of cervical cancer patients with radiologically negative lymph nodes treated under the same departmental planning and treatment delivery protocols using IMRT/VMAT (45 Gy in 25 fractions for EBRT and 28 Gy in 4 fractions for brachytherapy) without SIB were identified from the EMBRACE patients treated at this centre. The control group was matched with the SIB cohort for the length of follow up and histology to provide a ratio of 2 control over 1 SIB cases. Demographic and tumour characteristics between the treatment groups were compared using the Kruskal–Wallis test for continuous variables and Chi-square test for categorical variables.

Overall survival (OS) defined as death from any cause, local relapse free survival (LRFS) defined by relapse in the vagina, cervix, uterus, fallopian tubes or ovaries, regional relapse free survival (RRFS) defined by relapse in pelvic or para-aortic lymph nodes and distant relapse free survival (DRFS) defined by relapse in the peritoneal cavity, mediastinal or supraclavicular lymph nodes or distant organs including bone were calculated using the Kaplan Meier method; and the resulting survival curves compared using the Mantel-Cox log-rank test. For all tests, a *P* value < 0.05 was considered statistically significant. Statistical analysis was carried out with SPSS version 25.0 software (SPSS, Inc., Chicago, IL, USA).

## Results

Between January 2015 to December 2017, there were a total of 23 patients treated with SIB with a median follow up of 31.5 months (range 2.2–52.2). For the control cohort, 46 patients who received IMRT/VMAT without SIB were included with a median follow up of 30.2 months (range 1.4–79.3). The control group had no radiological evidence of nodal metastases. None of the patients were subject to laparoscopic node evaluation. Demographic details of patients in both groups are shown in Table 2. Apart from the length of follow up and histology, there were statistically significant differences in age and FIGO stages reflecting patients at younger ages and with more advanced FIGO staging being offered SIB.

**Table 2:** Patient demographic and characteristics of patients receiving external beam chemoradiotherapy (EBCRT) with and without simultaneous integrated boost (SIB) to radiologically positive lymph nodes

	Overall (n = 69)	EBRT with SIB (n = 23)	EBRT without SIB (n = 46)	*P* value
*Age*				
Median (years)	53.0	40.0	61.5	< 0.05
Range (years)	25–89	25–64	29–89	
*Follow up*				
Median (months)	30.9	31.5	30.2	0.52
Range (months)	1.4–79.3	2.2–52.2	1.4–79.3	
Histology				
Squamous carcinoma	53 (77%)	20 (87%)	33 (72%)	0.11
Adenocarcinoma	12 (17%)	1 (4%)	11 (24%)	
AdenoSquamous	4 (6%)	2 (9%)	2 (4%)	
*FIGO stage*				
IB	12 (17%)	2 (9%)	10 (22%)	< 0.05
IIA	10 (15%)	2 (9%)	8 (18%)	
IIB	36 (52%)	11 (48%)	25 (54%)	
IIIA	1 (1%)	1 (4%)	0	
IIIB	6 (9%)	5 (21%)	1 (2%)	
IVA	4 (6%)	2 (9%)	2 (4%)	

As shown in Table 3, 16/23 (70%) of the SIB cohort were staged with ^18^FDGPET and 5/23 (22%) were treated with SIB to positive para-aortic lymph nodes. 13/23 patients with FIGO I/II were upstaged to the FIGO IIIC1 due to the positive nodal disease. Within the SIB cohort, the average Dmean right pelvic nodal PTV was 67.8 Gy (62.4 from EBRT + 5.4 from Brachytherapy) and the average Dmean left pelvic nodal PTV dose was 67.7 Gy. (EBRT 62.4  from EBRT+  5.3 from Brachytherapy).

**Table 3:** Details of the staging and nodal involvement of patients receiving external beam chemoradiotherapy (EBCRT) with integrated boost (SIB) to radiologically positive lymph nodes

	Number of patients (%)
*Staging diagnostic imaging*	
CT/MRI	23 (100%)
FDG PET	16 (70%)
*Positive nodal regions*	
Pelvic Node	23 (100%)
Number of patients upstaged from the original FIGO I/II to revised FIGO IIIC1	13 (57%)
Para-aortic node	5 (22%)
Number of patients upstaged from the original FIGO I/II to revised FIGO IIIC2	2 (9%)
*Number of positive lymph nodes treated with SIB*	
1 node	6 (26%)
2 nodes	8 (35%)
3 nodes	2 (9%)
> 3 nodes	7 (30%)

All treated patients in the SIB cohort showed complete radiological response at the primary site on follow-up CT at 3 months. When considering the lymph nodes 83% of them had complete response three months after treatment and 13% showed partial response (> 50% regression). There were no recurrences at the primary site; two patients (2/23) with positive nodes relapsed at the treated nodal site; three-year LRFS was 90%. One of the two nodal relapses was in a patient presenting with massive adenopathy measuring 80 mm diameter.

**Fig. 1:**
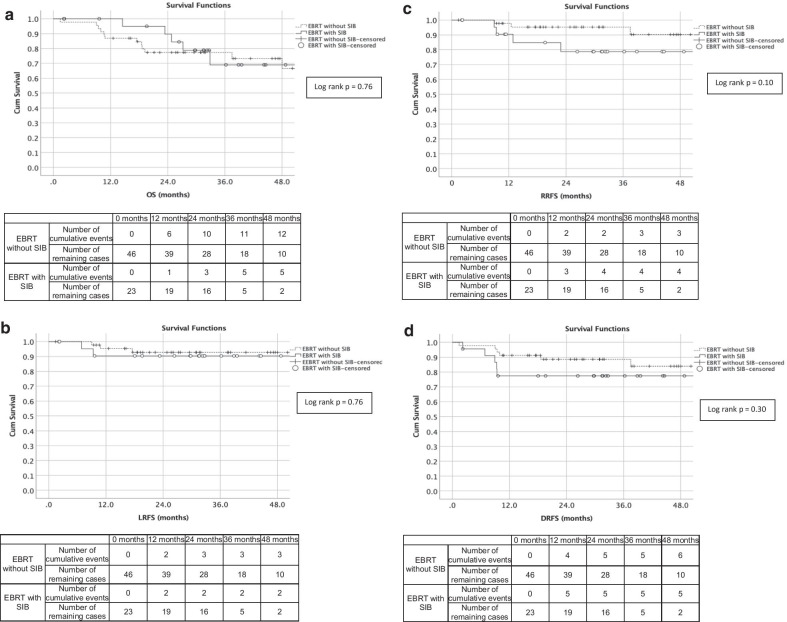
Kaplan–Meier overall survival (**a**), local recurrence free survival (**b**), regional recurrence free survival (**c**) and distant recurrence free survival free (**d**) curves for patients receiving cervical EBRT with and without simultaneous integrated boost to the nodal regions

The OS, LRFS, RRFS and DRFS at 3 years of the SIB cohort were 69%, 91%, 79% and 77% respectively compared to the control cohort, where these numbers were 77% (*p* = 0.76), 93% (*p* = 0.76), 95% (*p* = 0.10) and 89% (*p* = 0.30). As indicated and shown in Fig. 1, there were no statistically significant differences in the OS, LRFS, RRFS and DFRS rates between the SIB and control cohorts.

Acute and late toxicities are summarised in Table 4. No ≥ grade 3 gastrointestinal (GI) and genitourinary (GU) were found in the patients receiving EBRT with SIB.

**Table 4:** Acute and late post radiotherapy toxicities of patients receiving external beam chemoradiotherapy (EBCRT) with and without simultaneous integrated boost (SIB) to radiologically positive lymph nodes

	EBCRT with SIB (n = 23)	EBCRT without SIB (n = 46)	EBCRT with SIB (n = 23)	EBCRT without SIB (n = 46)
	Acute GI toxicities (%)		Acute GU toxicities (%)	
Grade 0	70	56	88	78
Grade 1	22	33	4	22
Grade 2	8	11	8	0
Grade 3	0	0	0	0
Grade 4	0	0	0	0
Grade 5	0	0	0	0
	Late GI toxicities (%)		Late GU toxicities (%)	
Grade 0	69	52	65	72
Grade 1	5	33	5	24
Grade 2	26	11	30	4
Grade 3	0	0	0	0
Grade 4	0	2	0	0
Grade 5	0	2	0	0
	Fatigue (%)		Vaginal stenosis (%)	
Grade 0	62	63	27	29
Grade 1	30	33	42	50
Grade 2	8	4	26	17
Grade 3	0	0	5	4
Grade 4	0	0	0	0
Grade 5	0	0	0	0
	Lymphoedema (%)		Pelvic fracture (%)	
Grade 0	79	94	95	85
Grade 1	17	4	5	11
Grade 2	4	2	0	4
Grade 3	0	0	0	0
Grade 4	0	0	0	0
Grade 5	0	0	0	0

## Discussion

The aim of this study was to review the results of a policy treating radiologically abnormal lymph nodes with a simultaneous integrated boost up to 60 Gy over 25 fractions in patients referred for radical chemoradiation in the setting of locally advanced cervical cancer.

Lymph node involvement has been regarded as an important prognostic factor in reports of conventionally treated cervical cancer patients; more than 50% of our SIB cohort were upstaged in FIGO staging due to positive lymph node involvement [5, 9, 11]. In this study, it is suggested that nodal involvement when treated to a high dose using the SIB technique did not adversely affect local control, distant disease-free survival or overall survival. Recent data from the EMBRACE study has shown that the low para-aortic nodes are the most common site of nodal relapse [9]. This is consistent with the positive results of RTOG 79-20 comparing the effect of prophylactic para-aortic irradiation [12] and the earlier EORTC study [13] which although it found no overall benefit suggested that in selected patients there may be a role for extended field irradiation. In contrast extended surgery to the para-aortic region has failed to show benefit [14].

An important result from this study is the finding that toxicity is not increased over that in a control cohort when SIB is used. No ≥ grade 3 toxicities in terms of acute and late GI/GU, fatigue, lymphoedema and pelvic fracture were found in our SIB cohort which is similar to other published studies [15–18]. Grade 3 vaginal stenosis was seen in 1 patient in both the control and the SIB group. It was noted that whilst overall there were similar rates of grade 1 and grade 2 late GU toxicities there were more grade 2 GU events in the SIB group and also grade 1 and 2 lymphoedema. Nearly one third of the SIB cohort received 60 Gy in 25 fractions to ≥ 3 lymph nodes which may account for this although with such small numbers in each group these observations are speculative only.

There are a limited number of published studies using SIB for nodal disease. The two largest series of 74 and 75 patients respectively report a good oncological outcome and a low toxicity profile [15, 19]. A series of 75 patients from the EMBRACE group delivered a median of 62 Gy EQD2 and reported 6 nodal failures with a median follow up of 30 months. The other series of 74 patients reported 3-year local control, distant metastasis-free survival, and overall survival rates of 91.7%, 75.7%, and 71.4% respectively [15]. These are compatible with our findings. In terms of treatment response of the lymph nodes receiving SIB, an excellent 96% partial or complete response rate was reported in our study. This is consistent with another study which reported a complete remission rate of 98.6% in a cohort of 23 node positive patients in which 74 nodes were treated by SIB delivering a dose of 55 Gy in the pelvis and 57.5 Gy in the common iliac and para-aortic regions [20].

However, the impact of a high dose boost to radiologically abnormal pelvic nodes remains uncertain and retrospective comparative data fails to confirm improvement in OS and LRFS in patients with locally advanced cervical cancer [10]. The results of this study do not suggest an major impact on overall survival or disease control being limited by the relatively small numbers. The results here however do show that a high rate of local control within abnormal lymph nodes can be delivered with low rates of toxicity. Whilst only a randomised trial would give robust evidence that the SIB contributed to this negating of the adverse effect of nodal metastases our results are suggestive that high doses to eradicate nodal disease within the pelvis and para-aortic nodes can be delivered without excessive toxicity.

Cervical cancer is a cancer which requires a high radiation dose for clinical and radiological remission of the primary tumour, and it would be expected that involved nodes may require a similar radiation dose for sustainable control. In this study the radiation dose to the SIB CTV was 67.8 Gy. This dose achieved complete remission and sustained nodal control. Dose response data from the EMBRACE study based on cumulative dose to the primary site combining external beam and brachytherapy doses has suggested that primary tumours < 3 cm require a dose of around 84 Gy EQD2_10_ [21]. In the EMBRACE series of 75 patients receiving an SIB for nodal disease a dose of 62 GyEQD2 (53–69 Gy EQD2) was given and no relation to dose or volume was seen in the 6 patients who relapsed [14]. Data from squamous carcinoma of the head and neck suggests that a dose of 65-70 Gy is adequate for control of nodal disease < 2 cm [22]. Several older series have assessed nodal relapse after definitive radiotherapy with increased dose to the nodes ranging from 52 Gy to 74.1 Gy [23–25]. At lower doses there was no dose response observed above 54 Gy but there is a suggestion that between 69.4 Gy and 74.1 Gy there may be a better control rate [24, 25]. However it should be noted that these trials used conformal two to four field techniques with sequential boosts and without the benefit of CT planning localisation of boosts and only one [25] included dose contribution from brachytherapy. This contrasts with the 68 Gy EQD2 achieved here in 6 weeks using multimodality imaging for localisation and including the brachytherapy contribution.

Limitations of this study are the small sample size and its retrospective nature. Reflecting the two cohorts with no randomised treatment allocation there were demographic differences between the treatment groups. The strength of this study is the use of a standardised radiotherapy treatment protocols and follow up procedures.

This data strongly supports the emerging picture that a high dose can be delivered to regional pelvic lymph nodes without excessive toxicity and with a high probability of local control. Whether this can alter the natural history for such patients and overcome the worse prognosis associated with positive lymph nodes in cervical cancer should be the subject of a multicentre prospective randomised trial to formally evaluate the role of SIB in this group of patients. In this it will be important to consider the impact of FDG PET staging which results in stage migration with many more node positive patients being found to have systemic metastases. With this in mind and reflecting on the impact of high radiation doses in releasing immunogenic antigens combination therapy using high dose radiation to macroscopic nodes with immunomodulating drugs may be the way forward.

## Data Availability

The data is not available on a public domain website; the investigators will provide the original data to bona fide investigators following appropriate data transfer agreements on request.

## References

[1] Bray F, Ferlay J, Soerjomataram I, Siegel R, Torre L, Jemal A (2018). Global cancer statistics 2018: GLOBOCAN estimates of incidence and mortality worldwide for 36 cancers in 185 countries. CA Cancer J Clin..

[2] National Institute for Health and Care Excellence (NICE) Cervival screening. https://cks.nice.org.uk/cervical-screening. Accessed 17 June 2020.

[3] National Comprehensive Cancer Network (US) NCCN clinical practice guidelines in oncology. Cervical cancer, version 1. 2017. Fort Washington, PA: National Comprehensive Cancer Network; 2017. 10.6004/jnccn.2017.0008 28040721

[4] Jürgenliemk-Schulz I, Beriwal S, de Leeuw A, Lindegaard J, Nomden C, Pötter R (2019). Management of nodal disease in advanced cervical cancer. Semin Radiat Oncol.

[5] Bhatla N, Berek J, Cuello Fredes M, Denny L, Grenman S, Karunaratne K (2019). Revised FIGO staging for carcinoma of the cervix uteri. Int J Gynecol Obstet.

[6] Cibula D, Potter R, Planchamp F, Avall-Lundqvist E, Fischerova D, Haie Meder C (2018). The European Society of Gynaecological Oncology/European Society for Radiotherapyand Oncology/European Society of Pathology Guidelines for the management of patients with cervical cancer. Int J Gynecol Cancer.

[7] Shumway J, Echeverria A, Patel U, Asper J, Bonnen M, Ludwig M (2017). Effectiveness of intensity-modulated radiation therapy with simultaneous integrated boost in cervical cancer patients with PET positive lymph nodes. J Radiat Oncol.

[8] Potter R, Tanderup K, Kirisits C, de Leeuw A, Kirchheiner K, Nout R (2018). The EMBRACE II study: the outcome and prospect of two decades of evolution within the GEC-ESTRO GYN working group and the EMBRACE studies. Clin Transl Radiat Oncol.

[9] Tan LT, Pötter R, Sturdza A, Fokdal L, Haie-Meder C, Schmid M (2019). Change in patterns of failure after image-guided brachytherapy for cervical cancer: analysis from the RetroEMBRACE study. Int J Radiat Oncol Biol Phys.

[10] Wujanto C, Choo B, Tan D, Ilancheran A, Ng J, Low J (2019). Does external beam radiation boost to pelvic lymph nodes improve outcomes in patients with locally advanced cervical cancer?. BMC Cancer.

[11] Taylor A, Rockall AG, Powell MEB (2007). An atlas of the pelvic lymph node regions to aid radiotherapy target volume definition. Clin Oncol (R Coll Radiol).

[12] Rotman M, Pajak TF, Choi K, Clery M, Marcial V, Grigsby PW (1995). Prophylactic extended-field irradiation of para-aortic lymph nodes in stages IIB and bulky IB and IIA cervical carcinomas ten-year treatment results of RTOG 79–20. JAMA.

[13] Haie C, Pejovic MH, Gerbaulet A, Horiot JC, Pourquier H, Delouche J (1988). Is prophylactic para-aortic irradiation worthwhile in the treatment of advanced cervical carcinoma? Results of a controlled clinical trial of the EORTC radiotherapy group. Radiother Oncol.

[14] Yoshida K, Kajiyama H, Yoshihara M, Ikeda Y, Yoshikawa N, Nishino K (2019). Does postoperative prophylactic irradiation of para-aortic lymph nodes reduce the risk of recurrence in uterine cervical cancer with positive pelvic lymph nodes?. Int J Clin Oncol.

[15] Dang Y-Z, Li P, Li J-P, Zhang Y, Zhao L-N, Li W-W (2019). Efficacy and toxicity of IMRT-based simultaneous integrated boost for the definitive management of positive lymph nodes in patients with cervical cancer. J Cancer.

[16] Cihoric N, Tapia C, Krüger K, Aebersold DM, Klaeser B, Lössl L (2014). IMRT with 18FDG-PET\CT based simultaneous integrated boost for treatment of nodal positive cervical cancer. Radiat Oncol.

[17] Marnitz S, Kohler C, Burova E, Wlodarczyk W, Jahn U, Grun A (2012). Helical tomotherapy with simultaneous integrated boost after laparoscopic staging in patients with cervical cancer: analysis of feasibility and early toxicity. Int J Radiat Oncol Biol Phys.

[18] Jethwa K, Jang S, Gonuguntla K, Evans J, Block M, Kumar A (2018). Lymph node-directed simultaneous integrated boost in patients with clinically lymph node-positive cervical cancer treated with definitive chemoradiation: clinical outcomes and toxicity. Int J Radiat Oncol Biol Phys..

[19] Ramlov A, Kroon PS, Jürgenliemk-schulz IM, de Leeuw AAC, Gormsen LC, Fokdal LU (2015). Impact of radiation dose and standardized uptake value of (18)FDG PET on nodal control in locally advanced cervical cancer. Acta Oncol.

[20] Lindegaard JC, Assenholt M, Ramlov A, Fokdal LU, Alber M, Tanderup K (2017). Early clinical outcome of coverage probability based treatment planning for simultaneous integrated boost of nodes in locally advanced cervical cancer. Acta Oncol.

[21] Tan LT, Tanderup K, Hoskin P, Cooper R (2018). Pötter R Image-guided adaptive brachytherapy for cervix cancer—a story of successful collaboration within the GEC-ESTRO GYN network and the EMBRACE studies. Clin Oncol (R Coll Radiol).

[22] Gomez PDJ, Rodriguez R, Rijo GJ, Alvarez I, Querejeta A, Alonso R (1992). Control of neck nodes in squamous cell carcinoma of the head and neck by radiotherapy: prognostic factors. Clin Otolaryngol.

[23] Small W, Winter K, Levenback C, Iyer R, Gaffney D, Asbell S (2007). Extended-field irradiation and intracavitary brachytherapy combined with cisplatin chemotherapy for cervical cancer with positive para-aortic or high common iliac lymph nodes: results of arm 1 of RTOG 0116. Int J Radiat Oncol Biol Phys.

[24] Grigsby PW, Singh AK, Siegel BA, Dehdashti F, Rader J, Zoberi I (2004). Lymph node control in cervical cancer. Int J Radiat Oncol Biol Phys.

[25] Rash DL, Lee YC, Kashefi A, Durbin-Johnson B, Mathai M, Valicenti R (2013). Clinical response of pelvic and para-aortic lymphadenopathy to a radiation boost in the definitive management of locally advanced cervical cancer. Int J Radiat Oncol Biol Phys.

