# Correction to: High dose simultaneous integrated boost for node positive cervical cancer

**DOI:** 10.1186/s13014-021-01887-2

**Published:** 2021-08-18

**Authors:** Iresha Jayatilakebanda, Yat Man Tsang, Peter Hoskin

**Affiliations:** 1 Mount Vernon Cancer Centre, Rickmansworth Road, Northwood, Middlesex, HA6 2RN UK; 2 University of Manchester, Manchester, UK

## Correction to: Radiat Oncol (2021) 16:92 https://doi.org/10.1186/s13014-021-01818-1

Following publication of the original article [1], the author identified an error in the author name of Iresha Jayatilakebanda.

- The incorrect author name is: Iresha Ayatilakebanda
- The correct author name is: Iresha Jayatilakebanda

The author group has been updated above and the original article [1] has been corrected.

## References

[1] Jayatilakebanda I, Tsang YM, Hoskin P (2021). High dose simultaneous integrated boost for node positive cervical cancer. Radiat Oncol.

